# Asthma in paediatric intensive care in England residents: observational study

**DOI:** 10.1038/s41598-022-05414-5

**Published:** 2022-01-25

**Authors:** Mome Mukherjee, Steve Cunningham, Mohammad Romel Bhuia, Tsz-Yan Milly Lo, Jasper V. Been, Aziz Sheikh

**Affiliations:** 1 Asthma UK Centre for Applied Research, Usher Institute, The University of Edinburgh, Edinburgh, UK; 2 HDR UK Better Care and BREATHE Hub, The University of Edinburgh, Edinburgh, UK; 3 Centre for Inflammation Research, Royal Hospital for Children and Young People, The University of Edinburgh, Edinburgh, UK; 4 Paediatric Critical Care Unit, Royal Hospital for Children and Young People, Edinburgh, UK; 5 Division of Neonatology, Department of Paediatrics, Department of Obstetrics and Gynaecology, and Department of Public Health, Erasmus MC–Sophia Children’s Hospital, University Medical Centre Rotterdam, Rotterdam, The Netherlands; 6 Division of General Internal Medicine and Primary Care, Brigham and Women’s Hospital/Harvard Medical School, Boston, MA USA

**Keywords:** Asthma, Paediatric research

## Abstract

Despite high prevalence of asthma in children in the UK, there were no prior report on asthma admissions in paediatric intensive care units (PICU). We investigated the epidemiology and healthcare resource utilisation in children with asthma presenting to PICUs in England. PICANet, a UK national PICU database, was queried for asthma as the primary reason for admission, of children resident in England from April 2006 until March 2013. There were 2195 admissions to PICU for a median stay of 1.4 days. 59% were males and 51% aged 0–4 years. The fourth and fifth most deprived quintiles represented 61% (1329) admissions and 73% (11) of the 15 deaths. Deaths were most frequent in 10–14 years age (n = 11, 73%), with no deaths in less than 5 years age. 38% of admissions (828/2193) received invasive ventilation, which was more frequent with increasing deprivation (13% (108/828) in least deprived to 31% (260/828) in most deprived) and with decreasing age (0–4-year-olds: 49%, 409/828). This first multi-centre PICU study in England found that children from more deprived neighbourhoods represented the majority of asthma admissions, invasive ventilation and deaths in PICU. Children experiencing socioeconomic deprivation could benefit from enhanced asthma support in the community.

## Introduction

Asthma is the most common chronic disease in children, with the prevalence higher in children in the UK than in the rest of Europe^1^. Asthma in UK children is responsible for substantial morbidity, with 2.8 million school days lost annually^2^, and more than 25,000 children admitted annually with asthma attacks^3^. Children’s asthma also directly impacts family members with 69% of parents or carers taking time off work and 13% give up their jobs completely to support their child’s asthma care^1^.

In children admitted to hospital in England, geographical and socio-economic status (SES) account for differences in asthma presentation and outcomes^4^. A single-centre study in the USA, which compared 161 paediatric intensive care units (PICU) to 610 non-PICU hospital admissions, reported that SES was not associated to PICU admission^5^. However, it was identified that among parents of the PICU admissions, 34% earned less than $15,000, 45% were educated up to high school level, 40% could not find work despite seeking employment, 38% had no resource for borrowing money in time of need, 74% did not own a home and 25% did not own a car^5^.

Admissions to paediatric intensive care with acute asthma are uncommon but may be considered a failure of asthma management and thus a bellwether of the general management of asthma in each community. A better understanding of the epidemiology and healthcare utilisation of children with acute asthma admitted to PICU may inform strategies to help reduce future asthma attack severity and mortality.

We describe the epidemiology and healthcare resource utilisation in children resident in England with asthma, who required admission to PICU.

## Methods

We interrogated electronic data collected prospectively between 1 April 2006 and 31 March 2013 of all PICU admissions of children resident in England, aged 0–14 years, who were given a primary diagnosis of asthma. The data includes children who had more than one admission in PICU for asthma. A protocol with a brief overview of methods has been published previously^6^. Asthma PICU admissions in England were compared to those in the whole of the UK and asthma deaths in PICU were compared to total deaths due to asthma as an underlying condition in England. We are reporting the study findings here following the REporting of studies Conducted using Observational Routinely-collected Data (RECORD) guideline^7^.

### Database

In the UK, the Paediatric Intensive Care Audit Network (PICANet)^8^, is a dedicated PICU database which enables common audit across sites. The database receives core government funding to support its use^9^. All UK PICU together with PICU in Ireland contribute data to PICANet^8^. We report results for England only due the small number of PICU admissions in Scotland, Wales and Northern Ireland. This means that information by SES was not available due to data confidentiality and that complete data over the study period was not available for these countries as they joined PICANet later. Since the data were originally obtained to estimate the cost of asthma to the public sector by financial year (i.e. 1 April to 31 March of next year)^6^, we included all National Health Service (NHS) PICU in England (excluding two private PICU) and retained the reporting convention by financial year, though PICANet data are reported annually from 1 January to 31 December each year. The number of English PICU contributing data varied over the study period due to addition of new PICU or merging of existing PICU. On average there were 25 PICU per year contributing to PICANet during the study period.

Some children who cannot be managed within the PICU of their resident UK-country receive care in PICU of another UK-country. Thus, we considered children whose country of residence was England even though they may have been treated elsewhere. Each child admitted to a participating PICU centre, provided information to the standardised PICANet dataset, including demographics, diagnosis, disease severity and clinical outcomes. Based on the findings recorded by intensivists, severity of illness in the first 24 h following PICU admission was auto-calculated based on an algorithm in the PICU electronic health record system, the ‘paediatric index of mortality’ score version 2 (PIM2), which was used in PICANet to standardise disease severity/care burden and to predict mortality in PICU^10^. The PIM2 mortality prediction model was an improvement of PIM version 1 and was developed using data at admission from 14 PICU in Australia, UK and New Zealand, using ten variables: (i) systolic blood pressure, (ii) pupillary reactions to bright light, (iii) partial pressure of oxygen (PaO2) (> 3 mm and both fixed = 1, other or unknown = 0), (iv) base excess in arterial or capillary blood, (v) mechanical ventilation at any time during the first hour in ICU (no = 0, yes = 1), (vi) elective admission to ICU (no = 0, yes = 1), (vii) recovery from surgery or a procedure is the main reason for ICU admission (no = 0, yes = 1), (viii) admitted following cardiac bypass (no = 0, yes = 1), (ix) high risk diagnosis—none/cardiomyopathy or myocarditis/cardiac arrest preceding ICU admission/hypoplastic left heart syndrome/severe combined immune deficiency/HIV infection/leukaemia or lymphoma after first induction/liver failure/spontaneous cerebral haemorrhage/neuro-degenerative disorder and x) low risk diagnosis—none or main reason for PICU admission was asthma/bronchiolitis/obstructive sleep apnoea/diabetic keto-acidosis^10^. PIM2 values ranged between 0 and 100%, where 0% meant no risk of death and 100% meant absolute risk of death**.** Neonatal ICU or high dependency care admissions do not form part of the PICANet dataset.

The SES of children admitted to PICU in England was derived by PICANet by linking the validated home address via the National Statistics Postcode Directory^11^, which has information on the English Index of Multiple Deprivation (EIMD). We used the most recent EIMD (2010) for our data in our analysis as we wanted to compare SES over time. EIMD 2010 was constructed based on weighted deprivation in each of the 38 indicators, which were then categorised into seven domains of income, employment, health, education, housing, crime and living environment, in each of the 3248 geographical areas in England, which are designed to be of a similar population size with an average of approximately 1500 or 650 households^12^. SES was categorised into quintiles: EIMD quintile 1 (EIMD1) represented the least deprived and EIMD5 the most deprived neighbourhood.

Deaths statistics for national and local regions published by the Office for National Statistics (ONS) were used to find all asthma deaths in England, in children aged 0–14 years, during the study period^13^.

### Study population and period

Only children who were aged under 15 years on the day of admission to PICU were included. Admissions with a primary diagnosis of asthma identified using Read codes version 3, published previously, were studied^6^.

### Data and analyses

Information are provided for financial years, by 5-year age-groups (0–4, 5–9 and 10–14 years), sex and socio-economic status. Healthcare resource utilisation was measured by ventilation types (invasive ventilation only, non-invasive ventilation only, or having received both invasive and non-invasive ventilation) and length of stay. Any form of ventilation support includes admissions which had ‘invasive ventilation only’ as well as ‘both invasive and non-invasive ventilation’. Outcome at PICU discharge was examined by survival. Number of deaths in PICU were compared to overall deaths for asthma, as an underlying condition, in under 15-year-olds in England.

Categorical data (age, sex, year, EIMD) were reported by counts and percentages, and continuous data (PIM2 and LoS) by median and inter-quartile range (IQR). The number of PICU admissions were age standardised using the English Standard Population. The denominators used were the respective mid-year population estimates for those age groups in June of that year. Age standardised rates and 95% confidence intervals (CI) with Poisson approximation^14^, were calculated per one million (1,000,000) population. The one-way ANOVA test was used to find if the average number of admissions were same in the EIMD quintiles. Since PIM2 and LoS were skewed, non-parametric tests of comparison were used for them. Differences in PIM2 and LoS for age-group, EIMD and year was performed using Kruskal–Wallis Chi-square test (H statistic, *p* value). Differences in PIM2 and LoS for sex was performed using the Mann–Whitney *U* test and Mantel–Haenszel Chi-square test for independence of ordinal categorical variables (EIMD, year). To find association of age, sex, EIMDs and year with PIM2 and LoS, separately, which were both positively skewed, age, sex and EIMDs were adjusted in regression models and finally a generalised linear model with gamma distribution and log link function was used, since it had the least Akaike Information Criterion. Multinomial logistic regression was used to find association between EIMDs and invasive ventilation, controlling for age, sex and year. We did not try to find factors that affected mortality, since we had a minimum dataset and did not have all the measurements taken in PICU. We only had years as a time measure, thus could not look at seasonality. Data were analysed using IBM SPSS Statistics version 25.

### Ethics

PICANet has approvals to collect patient-identifiable data without informed consent from Patient Information Advisory Group (now the NHS Health Research Authority Confidentiality Advisory Group) and ethics approval granted by the Trent Medical Research Ethics Committee, ref. 05/MRE04/17 + 5. Ethics approval for anonymised patient level data access was obtained from PICANet and from the University of Edinburgh’s Usher Institute’s Ethics Review Group^6^.

## Results

In the reporting period, 1 April 2006 to 31 March 2013, there were 2195 PICU admissions for asthma in children under 15 years of age resident in England. There were 78,615 children, including 15 years and above, admitted during the study period, of which 2110 children were for asthma (2.7%).

### Epidemiology

PICU admissions with a primary diagnosis of asthma are described in Table 1. The highest proportion of admissions were in those aged 0–4 years (51.1%, 1129/2195) with a male predominance (58.6%, 1286/2195). There was significantly higher risk of PICU admission for asthma in the 0–4-year-olds compared to the 5–9 and 10–14-year-olds (t statistics 7.5 (*p* < 0.001) and 6.2 (*p* < 0.001) respectively) and in the 5–9-year-olds compared to the 10–14-year-olds (t statistics − 2.2 (*p* = 0.02)). Only in the 0–4-year-olds, males had a significantly higher risk of PICU admission for asthma than females (t statistics 4.4 (*p* = 0.0004)).

**Table 1:** Admissions with asthma as the primary reason for admission in PICU in England with percentages by age-groups and males therein and age-standardised rate per million [number (n), percentages (%) and 95% confidence interval (95% CI)].

Asthma admissions		0–4 years		5–9 years		10–14 years		Age-standardised rate per million
	n	%	Male %	%	Male %	%	Male %	(95% CI)
2006–07	266	45.5	66.9	28.6	56.5	25.9	51.3	28.9 (28.4–29.5)
2007–08	296	49.3	59.6	26.4	55.6	24.3	47.4	31.9 (31.3–32.4)
2008–09	328	55.2	65.2	19.8	56.1	25.0	47.7	34.8 (34.2–35.4)
2009–10	326	54.9	63.7	17.2	56.0	27.9	48.2	34.3 (33.7–34.9)
2010–11	327	54.4	60.7	20.5	63.4	25.1	56.7	34.0 (33.4–34.6)
2011–12	283	45.9	60.0	24.4	58.3	29.7	59.4	29.6 (29.0–30.1)
2012–13	369	52.6	55.2	23.0	57.8	24.4	60.0	37.6 (37.0–38.2)
Overall	2195	51.1	61.6	22.8	57.7	26.0	53.0	

The estimated number of age standardised PICU admissions per million children in England, were 28.9 (95% CI 28.4–29.5) in 2006–07 and 37.6 (95% CI 37.0–38.2) in 2012–2013. Although the trend was positive (β = 0.74), it was not statistically significant.

### Severity

The PIM2 score was assessed as a marker of disease severity. PIM2 was less than 1% in 72.5% admissions and was over 30% in 0.4% admissions. Median PIM2 were similar in both girls and boys over the study period [median 0.4% (IQR 0.2–1.0%) vs 0.4% (IQR 0.2–1.1%) (H = 1.67, *p* = 0.196)]. The overall trend in PIM2 significantly decreased [4.4% (95% CI 2.3–6.5%; *p* = 0.000052)] when age, sex and EIMD were controlled (Supplementary Table S1, S2).

Compared to 0–4-year-olds, PIM2 when controlled for year and EIMD, was 1.13 times (95% CI 1.01–1.26, *p* = 0.031) significantly higher in 5–9-year-olds and 1.95 times (95% CI 1.74–2.19, *p* < 0.00001) significantly higher in 10–14-year-olds, indicating greater severity with increasing age (Supplementary Table S2, S3).

### Healthcare resource utilisation

*Mechanical ventilation* data was missing for two admissions (N = 2193) and LoS was missing in three admissions (N = 2192). These admissions are reported in overall numbers but excluded from mechanical ventilation and LoS analyses.

Table 2 shows the distribution of ventilation in asthma admissions in PICU in England.

**Table 2:** Number and percentages of paediatric admissions in England in the financial years in PICANet by ventilation provided.

	No ventilation		Only non-invasive ventilation		Only invasive ventilation		Received both invasive and non-invasive ventilation		Den**o**minator
	n	%	n	%	n	%	n	%	
2006–07	149	56.2	8	3.0	101	38.1	7	2.6	265
2007–08	174	59.0	12	4.1	101	34.2	8	2.7	295
2008–09	196	59.8	12	3.7	114	34.8	6	1.8	328
2009–10	208	63.8	*	*	103	31.6	*	*	326
2010–11	201	61.5	*	*	114	34.9	*	*	327
2011–12	157	55.5	8	2.8	112	39.6	6	2.1	283
2012–13	207	56.1	14	3.8	141	38.2	7	1.9	369
Overall	1292	58.9	73	3.3	786	35.8	42	1.9	2193

*One of those counts were less than 5, hence four are starred to prevent disclosure.

Over one-third of all admissions to PICU with asthma were mechanically ventilated (37.8%, 828/2193). A further 35.8% (95% CI 35.5–36.2; 786/2193) admissions received only invasive ventilation, while 1.9% (95% CI 1.9–2.0; 42/2193) received both invasive and non-invasive ventilatory support. Tracheostomy was performed in 0.7% of admissions. Any form of ventilation support was more commonly provided to 0–4-year-olds (49.4 (95% CI 46.0–52.8), 409/828), in males (62.3 (95% CI 59.0–65.6), 516/828) (Supplementary Table S3) and no difference was found over time (years).

*Length of stay in PICU:* Median LoS was 1.4 days (IQR 0.8–2.6) and remained much the same over the study period, when controlled for age, sex and EIMD (p = 0.31) (Supplementary Tables S1, S4). LoS was similar across sex and age groups: males median 1.4 (IQR 0.8–2.6), females 1.5 (0.9–2.6), 0–4 years 1.4 (0.8–2.6), 5–9 years 1.4 (0.8–2.8), 10–14 years 1.3 (0.8–2.5) days (Supplementary Table S3).

#### Socioeconomic status

The number of asthma PICU admissions increased with increase in deprivation (Table 3), which was irrespective of age (F = 37.2, *p* < 0.001).

**Table 3:** Severity and outcomes of PICU admissions with asthma as primary diagnosis or as underlying condition for death, by socio-economic status in England during 2006–2007 to 2012–2013.

	Number of admissions (%)	PIM2% (median, IQR)	Any form of ventilation support (%, 95% CI)	Length of stay (median, IQR)	Deaths [n (%)]
EIMD1 least deprived	249 (11.3)	0.5 (0.2–1.2)	108 (13.0 (10.7–15.3))	1.6 (0.8–2.5)	2 (13.3 (11.0–15.7))
EIMD2	251 (11.4)	0.6 (0.3–1.2)	117 (14.1 (11.8–16.5))	1.6 (0.9–2.6)	1 (6.7 (5.0–8.4))
EIMD3	366 (16.7)	0.4 (0.2–1.0)	139 (16.8 (14.2–19.3))	1.5 (0.8–2.6)	1 (6.7 (5.0–8.4))
EIMD4	505 (23.0)	0.5 (0.2–1.1)	204 24.6 ((21.7–27.6))	1.5 (0.8–2.8)	3 (20.0 (17.3–22.7))
EIMD5 most deprived	824 (37.5)	0.4 (0.2–1.0)	260 (31.4 (28.2–34.6))	1.3 (0.7–2.4)	8 (53.3 (49.9–56.7))
Overall	2195 (100.0)	0.4 (0.2–1.1)	828 (100.0)	1.4 (0.8–2.6)	15 (100)

After adjusting for age, sex and year, PIM2 score was 1.28 times higher (95% CI 1.10–1.49) in the least deprived areas, compared to those in the most deprived areas. Any form of ventilation support increased with increasing levels of deprivation (*p* = 0.000423): 13.0% in least deprived to 31.4% in most deprived (Table 3). In the multinomial regression, it was found that any form of ventilation, compared to no ventilation support was significantly less in females than in males (*p* = 0.007), in 0–4-year-olds (*p* = 0.01) compared to 10–14-year-olds and in most to least deprived areas (*p* = 0.001) (Supplementary Table S4).

LoS was not significantly different across SES in England (Supplementary Table S5): 1.3 vs 1.6 median days in the most deprived versus the least deprived (Table 3). After adjusting for year and EIMD, LoS was 1.14 times (95% CI 1.04–1.25, *p* = 0.006) higher in 10–14-year-olds compared to 0–4-year-olds and 9% lesser in males than in females (95% CI 2–15, p = 0.017) (Supplementary Table S5).

#### Survival

15 deaths occurred in children admitted to PICU with a primary diagnosis of asthma, with the majority in the 10–14 years olds group (73%, 11/15) and none in the pre-school group (Supplementary Table S3). Mortality rate was 0.7% (15/2195). Median PIM2 was higher amongst the non-survivors (31.7%, IQR 2.6–95.3%) compared to the survivors (0.4%, IQR 0.2–42.3%). Of the 15 who died, 14 (93.3%) required invasive ventilation. The one child who died without any ventilation support was in PICU for less than an hour. Children who died had a longer LoS than the survivors [median 2.9 (1.9–11.4) vs. 1.4 (0.8–23.8) days]. 60.7% admissions from the deprived neighbourhoods (EIMD 4, 5) contributed to 73% deaths (11/15) (Table 3). From ONS records, there were 127 deaths (as the underlying cause) in England, in under 15-year-olds, from asthma in the seven calendar years during 2006–2012. We found in the financial years 2006–2013, around 11.8% (15/127) of them were in PICU.

## Discussion

This is the first study profiling the epidemiology, healthcare resource utilisation and outcomes of asthma as the primary condition in children resident in England admitted in PICU, found that children from deprived neighbourhoods (EIMD 4, 5) comprised most of the admissions (61%), received mechanical ventilation most frequently (60%) and suffered the highest proportion of deaths (75%). Deaths were more common in 10–14-year-olds. PICU admission for asthma is infrequent and there was no clear trend. Most admissions and mechanical ventilation occur in preschool children. Deaths in PICU represented only 12% of all deaths due to asthma over the years investigated.

The PICANet dataset used here is of high quality from a national audit network on PICU in the UK, with high data completeness for England. Sample selection bias is minimised because PICANet covers the entire childhood population admitted to PICU in England. Limitations of the study were: (i) asthma is often difficult to diagnose in children under five years of age. It is quite probable that admissions coded as asthma represented other acute wheezing conditions, including acute viral induced wheeze^15,16^. Preschool admissions with a diagnosis of asthma were higher than those reported from other countries^5,17–25^. (ii) Due to confidentiality issues coupled with small numbers from Scotland, Wales and Northern Ireland or the PICU asthma deaths in England, detailed analyses from these episodes were not possible, thus limiting our ability to comment on reasons for regional variations or deaths^26^. (iii) PICANet dataset does not capture children admitted to adult ICU for asthma treatment. This number is considered small because children admitted into adult ICU have been referred to PICU in the UK since 1996^27^. (iv) The data of PICU admissions do not furnish us with the number of childhood patients. Thus, if a child had multiple admissions, that would inflate the association with SES. (v) Since the data were originally obtained to study the burden of asthma^2^, we did not collect clinical data such as arterial blood gases, blood cell count, chest X ray, lung function, etc. (vi) Asthma was a low-risk diagnosis in PIM2. We did not have access to predictive severity score which is asthma specific, for example the initial Modified Pulmonary Index Score (MPIS)^28^. (vii) Our data pertained to all admissions during PICU stay only and did not include pre or post-hospitalisations. Furthermore, some patients may have been admitted more than once in the year. Thus we cannot compare to findings where frequency and time to PICU re-admissions were found to be similar in Canada and USA and a higher proportion of children were readmitted to PICU in USA^29^.

Whilst asthma related PICU deaths in England (0.7%) were similar to those found in Australia (0.6%) and the Netherlands (0.6%)^17,18^, the reporting of asthma mortality in multi-centre PICU studies in the USA varied (0.3%, 1.9%, 4.3%)^19,21,30^. A UK study on general admissions in PICU using PICANet, found children who died in PICU stayed longer than who survived^31^, similar to our study. Given the small number of asthma deaths, we could not find if there was a reduction in asthma deaths in England's PICU over time. One UK study on all paediatric admissions in adult ICU reported that crude mortality fell from 6.7 in 1996 to 2.8% in 2011 (overall 4.6%)^26^. The reduction in PICU deaths was ascribed to development of PICU in the UK from its infancy in the nineties, whereby many children who would have been admitted to adult ICU by mid 2000s were admitted to PICU or referred promptly from adult ICU to PICU^26^. Thus the observed fall in percentage might not be a true trend. Most deaths from asthma in children in England are outside PICU (88%), as also observed in Australia (78%) and Ireland (100%)^32,33^.

Our previous study had found that although prevalence of severe asthma and hospitalisations were higher in children in lower SES, asthma deaths in the community were higher in children from higher SES^4^. This study, however, finds that asthma deaths in PICU were higher in children from lower SES. Although it is difficult to find ascribe clear reasons for this anomaly, it could be speculated that there are some psychosocial processes that contribute to this^34^. Air pollution and parental stress were found to be associated to increased childhood asthma, which was more prevalent in children living in lower SES areas^35^. The National Review of Asthma Deaths (NRAD) had reported that two-third of the asthma deaths could be preventable if they were attentively managed in primary care^3^. Identifying children in whom a tailored asthma management may prevent PICU admission could target those with asthma symptoms for over a week, older and/or exposed to tobacco smoke^36^.

The other considerable consequence of a PICU admission is the emotional burden in individuals who needed intensive care and their parents and carers, irrespective of survival^37–41^. This, and the decline in pulmonary function after PICU discharge^42^, highlight that healthcare professionals need to understand the entire patient journey from asthma diagnosis, asthma exacerbation to discharge, so that patients are better managed and the need for a PICU admission does not arise^43–45^. Since asthma triggers and symptoms vary in individuals, it is important to consider the family in communications and decision making^43–45^.

Use of mechanical ventilation in children admitted to PICU in England (37.8%) is higher than that found in most other multi-centre reports from Australia (6–14%), the Netherlands (19%), while it varied in USA (19%, 3–47%)^17,18,21,46^. This may reflect a lower threshold for PICU admission in some countries, i.e. increasing the denominator. Unlike our study, a multi-centre study in the Netherlands found a trend for increasing mechanical ventilation use over time^18^, with an overall levels of half of those that we report (36% vs. 19%)^47^. The length of PICU stay they reported was double that we report (median 1.4 vs. 3 days) but with similar mortality rates (0.7% vs. 0.6%)^18^, suggesting a lower threshold for admission. The male preponderance found in our study concurs with the literature^5,17–24,46,48,49^.

This is the first study utilising data from multiple sites to report the association of SES on asthma in PICU admissions. The population distribution of under-15-year-olds in England in 2010 was 24.0% in most deprived neighbourhoods and 19.3% in least deprived neighbourhoods. We found 37.5% admissions from most deprived neighbourhoods and 11.3% from least deprived neighbourhoods. Unlike a single-centre study in USA^5^, we did find association of deprivation to PICU admissions, length of stay and deaths. Furthermore, this is also the first study that found that asthma severity, as determined by adjusted PIM2 score, could be higher in children from higher SES.

This study highlights an urgent need to identify ways to improve asthma outcomes for children, particularly in deprived communities. Addressing the causes of acute asthma exacerbations through education and appropriate treatment should lower PICU admissions. Ideally this should be delivered within primary care, especially in difficult to reach communities where access to primary care is limited.

In addition to clinical treatment, there remains an unmet need for novel therapeutics to alleviate severe cases of acute asthma exacerbations. Little is known about the efficacy of biologic therapies in paediatric asthma, their optimum duration for treatment and potential long term side effects into adulthood^50–53^. Thus the Global Initiative for Asthma (GINA) recently published considerations for selecting biologic therapy for severe asthma in children younger than 18 years is helpful^54^.

A focus on asthma education of children, parents and caregivers to act early in preventing acute asthma exacerbations from progressing should be a key objective to reduce both hospital and PICU admissions. Such education can be delivered across social and healthcare settings and needs to be repeated regularly to ensure good understanding and ability to respond when needed.

## Conclusions

In this first study of data across multiple sites in England we found that there are over 300 PICU admissions in the critically ill children with asthma in a year and children from more deprived neighbourhoods had most of the asthma admissions, invasive ventilation and deaths in PICU. Reducing severe asthma attacks will require a coherent, consistent and cross-disciplinary approach (healthcare professionals, schools, third sector) to raise awareness of severe asthma and how children and parents/caregivers can reduce its impact.

## Supplementary Information

Supplementary Information.

## Data Availability

The data that support the findings of this study are available from PICANet (https://www.picanet.org.uk/data-collection/data-requests/) but restrictions apply to the availability of these data and so are not publicly available. Analysed data are presented in the paper and supplementary file.
