# Supplementary material for: Asthma in paediatric intensive care in England residents: observational study
Source: Sci Rep. 2022 Jan 25;12:1315. doi: 10.1038/s41598-022-05414-5 (PMC8789863; doi:10.1038/s41598-022-05414-5)
Supplement: Supplementary file 1 — Supplementary Information. [file 41598_2022_5414_MOESM1_ESM.docx]

**Table S1: Severity by PIM2 score, any form of ventilation support and length of stay in the financial years in England for asthma in PICANet**

| **England** | **PIM2** median percentage (IQR) | **Any form of ventilation support** (n, % (95%CI)) | | **Length of stay**  median days (IQR) |
| --- | --- | --- | --- | --- |
| 2006-07 | 0.6 (0.3-1.3) | 108 | 40.8 (34.8-46.7) | 1.3 (0.7-2.7) |
| 2007-08 | 0.4 (0.2-1.2) | 109 | 36.9 (31.4-42.5) | 1.5 (0.7-2.5) |
| 2008-09 | 0.4 (0.3-1.1) | 120 | 36.6 (31.4-41.8) | 1.3 (0.7-2.6) |
| 2009-10 | 0.3 (0.2-1.0) | 109 | 33.4 (28.3-38.6) | 1.4 (0.8-2.6) |
| 2010-11 | 0.4 (0.2-1.0) | 116 | 35.5 (30.3-40.7) | 1.6 (0.9-2.6) |
| 2011-12 | 0.5 (0.3-1.0) | 118 | 41.7 (36.0-47.4) | 1.5 (0.9-2.5) |
| 2012-13 | 0.5 (0.2-1.1) | 148 | 40.1 (35.1-45.1) | 1.5 (0.9-2.7) |

**Table S2: Adjusted ratios of PIM2 scores estimated using generalised liner regression model with gamma distribution and log link function**

| **Characteristics** | **Ratio of PIM2 score (95% CI)** | **P-value** |
| --- | --- | --- |
| ***Sex*** |  | -  0.054 |
| Female | 1 |  |
| Male | 0.912 (0.83-1.002) |  |
| ***Age*** |  |  |
| 0-4 years | 1 | - |
| 5-9 years | 1.13 (1.01-1.26) | 0.031 |
| 10-14 years | 1.95 (1.74-2.19) | <0.001 |
| ***Year*** | 0.96 (0.94-0.98) | <0.001 |
| ***EIMD*** |  |  |
| EIMD1 most deprived | 1 | - |
| EIMD2 | 1.03 (0.92-1.16) | 0.596 |
| EIMD3 | 0.77 (0.68-0.88) | <0.001 |
| EIMD4 | 0.98 (0.84-1.14) | 0.769 |
| EIMD5 least deprived | 1.28 (1.10-1.49) | 0.002 |

**Table S3**: **PIM2 score, length of stay, any form of ventilation support and deaths in paediatric admissions in England in PICANet by age-groups and sex**

| **Age-groups**  **sex** | **PIM2 score**  median % (IQR) | **Any form of ventilation support** % admissions (95% CI) | **Length of stay**  median days (IQR) | **Deaths** |
| --- | --- | --- | --- | --- |
| 0-4 years | 0.4 (0.2-1.0) | 49.4 (46.0-52.8) | 1.4 (0.8-2.6) |  |
| Males vs Females | 0.4 (0.2-1.0) vs  0.4 (0.2-1.0) | 60.4 (55.7-65.1) vs 39.6 (34.9-44.3) | 1 .3(0.7-2.6) vs  1.5 (0.9-2.8) |  |
| 5-9 years | 0.4 (0.2-1.0) | 26.0 (23.0-29.0) | 1.4 (0.8-2.8) | 4 (26.7 (4.3-49.0)) |
| Males vs Females | 0.4 (0.3-1.0) vs  0.4 (0.2-1.0) | 62.3 (55.8-68.8) vs 37.7 (31.2-44.2) | 1.4 (0.8-2.4) vs  1.5 (0.8-2.4) | 1 (25 (-17.4-67.4)) vs  3 (75 (32.6-117.4)) |
| 10-14 years | 0.5 (0.3-1.2) | 24.6 (21.7-27.6) | 1.3 (0.8-2.5) | 11 (73.3 (51-95.7)) |
| Males vs Females | 0.8 (0.3-1.3) vs  0.4 (0.2-1.1) | 66.2 (59.7-72.7) vs 33.8 (27.3-40.3) | 1.5 (0.9-2.8) vs  1.5 (0.8-2.6) | 7 (63.6 (35.2-92.1)) vs  4 (36.4 (7.9-64.8)) |
| Overall | 0.4 (0.2-1.1) | 37.8 (34.5-41.1) | 1.4 (0.8-2.6) | 15 (100 (100-100) |
| Males vs Females | 0.4 (0.2-1.1) vs  0.4 (0.2-1.0) | 62.3 (59.0-65.6) vs 37.7 (34.4-41.0) | 1.4 (0.8-2.6) vs  1.5 (0.9-2.6) | 7 (53.3 (28.1-78.6)) vs  8 (46.7 (21.4-71.9)) |

**Table S4: Adjusted ratios of any form of ventilation support compared to no ventilation,** **using multinomial regression**

| **Characteristics** | **Ratio of invasive ventilation only to no ventilation (95% CI)** | **P-value** |
| --- | --- | --- |
| ***Sex*** |  |  |
| Male | 1 | - |
| Female | 0.78 (0.65-0.93) | 0.007 |
| ***Age*** |  |  |
| 10-14 years | 1 | - |
| 5-9 years | 0.80 (0.62-1.03) | 0.08 |
| 0-4 years | 0.75 (0.60-0.94) | 0.01 |
|  |  |  |
| ***EIMD*** |  |  |
| EIMD5 least deprived | 1 | - |
| EIMD4 | 1.11 (0.78-1.60) | 0.54 |
| EIMD3 | 0.81 (0.58-1.13) | 0.22 |
| EIMD2 | 0.91 (0.67-1.25) | 0.57 |
| EIMD1 most deprived | 0.60 (0.45-0.81) | 0.001 |

**Table S5: Adjusted ratios of length of stay estimated using generalised liner regression model with gamma distribution and log link function**

| **Characteristics** | **Ratio of length of stay (95% CI)** | **P-value** |
| --- | --- | --- |
| ***Sex*** |  |  |
| Female | 1 | - |
| Male | 0.91 (0.85-0.98) | 0.017 |
| ***Age*** |  |  |
| 0-4 years | 1 | - |
| 5-9 years | 0.91 (0.83-0.10) | 0.042 |
| 10-14 years | 1.14 (1.04-1.25) | 0.006 |
| ***Year*** | 0.99 (0.97-1.01) | 0.317 |
| ***EIMD*** |  |  |
| EIMD1 most deprived | 1 | - |
| EIMD2 | 1.08 (0.98-1.19) | 0.127 |
| EIMD3 | 0.98 (0.87-1.09) | 0.649 |
| EIMD4 | 1.04 (0.92-1.18) | 0.547 |
| EIMD5 least deprived | 1.02 (0.90-1.15) | 0.797 |
